# Hypoxic condition induced H3K27me3 modification of the LncRNA Tmem235 promoter thus supporting apoptosis of BMSCs

**DOI:** 10.1007/s10495-022-01747-8

**Published:** 2022-07-02

**Authors:** Fei Zhang, Hong Luo, Wuxun Peng, Lei Wang, Tao Wang, Zhihong Xie, Jian Zhang, Wentao Dong, Xiaohan Zheng, Gang Liu, Xuesong Zhu, Qinglin Kang, Xiaobin Tian

**Affiliations:** 1 Department of Orthopedics, The Affliated Hospital of Guizhou Medical University, Guiyang, 550004 Guizhou China; 2 School of Clinical Medicine, Guizhou Medical University, Guiyang, 550004 Guizhou China; 3 Department of Orthopedics, The First Affliated Hospital of Soochow University, Suzhou, 215000 Jiangsu China; 4 Department of Orthopedics, The Sixth People’s Hospital Affiliated to Shanghai Jiaotong University, Shanghai, 200233 China

**Keywords:** H3K27me3, BMSCs, Apoptosis, Hypoxia, Long non-coding RNA

## Abstract

Bone marrow mesenchymal stem cells (BMSCs) have strong regenerative potential and show good application prospects for treating clinical diseases. However, in the process of BMSC transplantation for treating ischemic and hypoxic diseases, BMSCs have high rates of apoptosis in the hypoxic microenvironment of transplantation, which significantly affects the transplantation efficacy. Our previous studies have confirmed the key role of long non-coding RNA Tmem235 (LncRNA Tmem235) in the process of hypoxia-induced BMSC apoptosis and its downstream regulatory mechanism, but the upstream mechanism by which hypoxia regulates LncRNA Tmem235 expression to induce BMSC apoptosis is still unclear. Under hypoxic conditions, we found that the level of LncRNA Tmem235 promoter histone H3 lysine 27 trimethylation modification (H3K27me3) was significantly increased by CHIP-qPCR. Moreover, H3K27me3 cooperated with LncRNA Tmem235 promoter DNA methylation to inhibit the expression of LncRNA Tmem235 and promote apoptosis of BMSCs. To study the mechanism of hypoxia-induced modification of LncRNA Tmem235 promoter H3K27me3 in the hypoxia model of BMSCs, we detected the expression of H3K27 methylase and histone demethylase and found that only histone methylase enhancer of zeste homolog 2 (EZH2) expression was significantly upregulated. Knockdown of EZH2 significantly decreased the level of H3K27me3 modification in the LncRNA Tmem235 promoter. The EZH2 promoter region contains a hypoxia-responsive element (HRE) that interacts with hypoxia-inducible factor-1alpha (HIF-1α), which is overexpressed under hypoxic conditions, thereby promoting its overexpression. In summary, hypoxia promotes the modification of the LncRNA Tmem235 promoter H3K27me3 through the HIF-1α/EZH2 signaling axis, inhibits the expression of LncRNA Tmem235, and leads to hypoxic apoptosis of BMSCs. Our findings improve the regulatory mechanism of LncRNA Tmem235 during hypoxic apoptosis of BMSCs and provide a more complete theoretical pathway for targeting LncRNA to inhibit hypoxic apoptosis of BMSCs.

**Supplementary Information:**

The online version contains supplementary material available at 10.1007/s10495-022-01747-8.

## Introduction

Bone marrow mesenchymal stem cells (BMSCs) are non-hematopoietic stem cells with self-renewal ability and multiple differentiation potentials [1–3]. Major breakthroughs have been made in the use of BMSCs to solve neurological diseases, meniscus damage repair, and hematological diseases [4–6]. However, the transplantation effect of BMSCs is less obvious for treating ischemic and hypoxic diseases, such as avascular necrosis of the femoral head [7–9]. We used BMSC transplantation to repair the model of avascular necrosis of the femoral head in the early stage, but the transplantation effect was unsatisfactory. In such diseases, the clinical application of BMSCs still has many issues that remain to be solved. Indeed, the transplanted BMSCs have high rates of apoptosis in the hypoxic environment of the lesion area, which greatly limits the transplantation efficacy of BMSCs. Therefore, identifying and developing methods to inhibit the hypoxic apoptosis of BMSCs are key to further improve the therapeutic effect of BMSCs in hypoxic ischemic diseases. At present, the main methods to inhibit the apoptosis of BMSCs include drug pretreatment, improvement of mitochondrial function, scavenging of excess reactive oxygen species (ROS), and inhibition of apoptosis-related proteins. However, the effects of these methods are still unsatisfactory [7, 10]. Therefore, it is of great practical significance to identify new targets and develop new methods to inhibit hypoxic apoptosis of BMSCs.

Studies have found that although long non-coding RNAs (LncRNAs) cannot directly encode proteins, they can regulate the expression of apoptosis-related genes before, during, and after gene transcription, thereby interfering with apoptosis [11–14]. To determine which LncRNAs are related to the hypoxic apoptosis of BMSCs, we used LncRNA chip combined with bioinformatics analysis. Our results showed that LncRNA transmembrane protein 235 (LncRNA Tmem235) may be involved in the hypoxic apoptosis of BMSCs. Our previous studies have demonstrated the key role of LncRNA Tmem235 in the hypoxia-induced apoptosis of BMSCs and its downstream regulatory mechanism; that is, LncRNA Tmem235 can interact with the apoptosis inhibitor baculoviral inhibitor of apoptosis [IAP] repeat-containing 5 (BIRC5) and competitively bind microRNA 34a-3p (miR-34a-3p) to reduce the inhibitory effect of miR-34a-3p on BIRC5, thereby promoting the expression of BIRC5. Finally, BIRC5 inhibits the activity of apoptosis-enforcing proteins, such as cysteinyl aspartate-specific proteinase-3/9 (CASP-3/9), thereby inhibiting the hypoxic apoptosis of BMSCs. However, in hypoxia-treated BMSCs, down-regulation of LncRNA Tmem235 expression reduces the anti-apoptotic effect mediated by the miR-34a-3p/BIRC5 pathway, which in turn promotes hypoxic apoptosis of BMSCs. However, the upstream mechanism by which hypoxia-regulated LncRNA Tmem235 expression induces BMSC apoptosis remains unclear.

Histone methylation is an important epigenetic modification that can regulate gene expression and mainly occurs at the lysine residues of histones [15, 16]. The trimethylation of histone H3 lysine 27 (H3K27me3) modification is mainly regulated dynamically by intracellular lysine methyltransferases and demethylases [17, 18]. The methylation modification of H3K27 is mainly regulated by polycomb repressive complex 2 (PRC2), the core catalytic unit of which is the enhancer of zeste homolog 2 (EZH2). Moreover, the C-terminal (C-ter) SET domain of PRC2 catalyzes the methylation of H3K27 to H3K27me3 [19–21]. Moreover, the lysine demethylase 6 (KDM6) family of proteins mediates H3K27me3 demethylation, such as that of KDM6A and KDM6B [22, 23]. DNA methylation is another important epigenetic modification that can cooperate with H3K27me3 modification to regulate gene expression [24, 25]. Methylated DNA binds to DNA-binding proteins, resulting in a highly compact arrangement of the bound DNA strands, which affects the binding of transcription factors and RNA polymerases to DNA strands and inhibits gene expression [26, 27]. To this end, we speculated that the expression of LncRNA Tmem235 may be related to H3K27me3 modification and DNA methylation. Hypoxia-inducible factor-1alpha (HIF-1α), as a hypoxia transcriptional activator, can bind to the hypoxia-responsive element (HRE) of a gene to activate gene expression. The promoter region of EZH2 also contains an HRE [28–30]. Therefore, under hypoxic conditions, HIF-1α may interact with EZH2 and participate in the modification of H3K27me3 in the promoter region of LncRNA Tmem235, thereby regulating the expression of LncRNA Tmem235 to affect the hypoxic apoptosis of BMSCs.

Based on the previous research results, we further studied the mechanism of hypoxia inhibiting the expression of LncRNA Tmem235, which is the expansion and extension of the downstream regulatory mechanism of LncRNA Tmem235 in the process of hypoxia-induced apoptosis of BMSCs, providing a more complete theory for ways in which to inhibit the hypoxic apoptosis of BMSCs by targeting LncRNA.

## Experiment section

### Animal

Twenty male Sprague Dawley rats, male or female, 10.0–20.0 g, were used in this study, and these were provided by the Animal Experiment Center of Guizhou Medical University. All experiments on rats were performed according to the guidelines of the Animal Care Welfare Committee of Guizhou Medical University.

### Acquisition and culture of BMSCs

The bilateral femur and tibia of SD rats were removed under sterile conditions, and the medullary cavity was washed with complete L-DMEM medium to obtain the bone marrow tissue. The bone marrow tissue was collected by centrifugation (2000 rpm, 2 min), resuspended in complete L-DMEM medium, and inoculated into cell culture flasks for culture. When the primary cells covered the bottom of the culture flask, subculture was performed at a ratio of 1:3.

### Osteogenic differentiation of BMSCs

Passage 2 BMSCs were seeded in six-well plates. When the cell fusion rate at the bottom of the six-well plate reached 60%, according to the instructions of BMSC osteogenic induction kit (Cyagen Biosciences, USA), the control group was maintained in complete L-DMEM medium, and the experimental group was cultured with BMSC osteogenic induction medium. After 2 weeks of osteogenic induction, the cells were fixed and alkaline phosphatase (ALP) activity was detected by ALP (Cyagen Biosciences, USA) staining.

### Adipogenic differentiation of BMSCs

Passage 2 BMSCs were seeded in six-well plates. When the cell fusion rate at the bottom of the six-well plate reached 60%, according to the instructions of BMSC adipogenic induction kit (Cyagen Biosciences, USA), the control group was maintained in complete L-DMEM medium, while the experimental group was cultured with BMSC adipogenic induction medium. After 3 weeks of adipogenic induction, staining was performed with Oil Red O (Cyagen Biosciences, USA).

### Chondrogenic differentiation of BMSCs

Passage 2 BMSCs were seeded in six-well plates. When the cell fusion rate at the bottom of the six-well plate reached 60%, according to the instructions of BMSC chondrogenesis induction kit (Cyagen Biosciences, USA), the control group was kept in complete L-DMEM medium, while the experimental group was cultured with BMSC chondrogenesis induction medium. After 4 weeks of chondrogenesis induction, acid mucopolysaccharide was identified in the cartilage tissue by Alcian blue (Cyagen Biosciences, USA) staining.

### Surface antigen identification of BMSCs

The density of passage 2 BMSCs was adjusted to 2 × 10^7^ cells/mL. In the single label group, after adding 5 μL of APC mouse anti-rat CD11b (BD, USA), PE-Cy™7 mouse anti-rat CD45 (BD, USA), V450 mouse anti-rat CD29 (BD, USA), PE mouse anti-rat CD90 (BD, USA), and PE mouse anti-rat CD106 (BD, USA) to each flow detection tube, 45 of μL staining buffer was added. For the multi-color labeling group, 5 µL of each of the above antibodies were added to a flow detection tube, followed by 25 μL of staining buffer. For the blank control, only 50 μL of staining buffer was added. Next, 50 μL of cell suspension was added to each tube, mixed well, incubated at 4 °C for 0.5 h protected from light, resuspended, and washed twice with staining buffer. Finally, 500 μL of staining buffer was added to each tube and detected by flow cytometry.

### BMSC hypoxia model

When the density of the second generation BMSCs reached 60%, they were divided into the following four groups according to different oxygen concentration treatments: normoxia (21% O_2_, 74% N_2_, and 5% CO_2_), 5% O_2_ group (5% O_2_, 90% N_2_, and 5% CO_2_), 1% O_2_ group (1% O_2_, 94% N_2_, and 5% CO_2_), and hypoxia (0% O_2_, 95% N_2_, and 5% CO_2_). BMSCs were cultured for 48 h under different oxygen concentration conditions for subsequent experiments.

### Mitochondrial membrane potential

When the fusion rate of the second-generation BMSCs treated with different oxygen concentrations reached 90%, a working solution of JC-1 was prepared according to the instructions of the mitochondrial membrane potential detection kit (KeyGEN BioTECH, China). After washing the cells with phosphate buffered saline (PBS), 1 ml of JC-1 working solution was added, before incubating the cells in an incubator for 0.5 h. Next, the cells were washed twice with 1 × incubation buffer, before adding 1 ml of complete L-DMEM medium and observing under a laser confocal microscope (Zeiss, Germany).

### ATP detection

According to the instructions of the ATP Colorimetric Assay Kit (Abcam), 1 × 10^6^ s-generation BMSCs treated with different oxygen concentrations were dissolved in 100 μL of ATP Assay Buffer and centrifuged at 4 °C and 15,000 g for 8 min. The collected supernatant was deproteinized with the Deproteinizing Sample Preparation Kit—TCA (Abcam) to remove interfering enzymes. Next, 5 μL of enzyme-depleted supernatant was added to a 96-well transparent plate and made up to 25 μL/well with the ATP Assay Buffer. The standard well included 25 μL/well of standard dilution. Next, 22 μL of the ATP Assay Buffer was mixed with 1 μL of ATP Probe, 1 μL of ATP Converter, and 1 μL of Developer Mix into a 25-μL Reaction Mix and added to each well. The samples were mixed well and incubated at room temperature for 0.5 h protected from light before measuring the absorbance at an optical density (OD) of 570 nm.

### ROS detection

After the second passage, BMSCs were treated with different oxygen concentrations, before washing with PBS and staining according to the instructions of the DCFDA/H2DCFDA—Cellular ROS Assay Kit (Abcam). Green fluorescence was observed after the cells had been incubated in the incubator for 0.5 h.

### Western blot

RIPA cell lysate was used to extract cellular proteins, and the BCA Protein Assay Kit (Abcam) was used to quantify the proteins. The SDS-PAGE gel was prepared, and 6 μL of denatured protein was added to each well for electrophoresis and transferred to a PVDF membrane. The primary antibodies were as follows: rabbit anti-β-actin (4970, Cell Signaling), rabbit anti-cleaved CASP-3 (ab32042, Abcam), rabbit anti-BIRC5 (ab196495, Abcam), rabbit anti-CASP-9 (ab2324, Abcam), rabbit anti -EZH2 (49–1043, Invitrogen), rabbit anti-SMYD3 (ab187149, Abcam), rabbit anti-KDM6A (ab253183, Abcam), rabbit anti-KDM6B/JMJD3 (ab169197, Abcam), rabbit anti-ESET (2196, Cell Signaling), and rabbit anti-histone H3 (tri methyl K27) (ab192985, Abcam). Goat anti-rabbit IgG H&L (HRP) (ab6721, Abcam) was used as the secondary antibody. Immobilon Western Chemiluminescent HRP Substrate (Millipore) was used for exposure; images were acquired using a gel imaging system, and quantitative analysis was performed by ImageJ.

### Apoptosis detection of BMSCs

TUNEL staining solution (Beyotime, China) was added to fixed and permeable second-generation BMSCs, and the cells were incubated at 37 °C in the dark for 60 min; the cells were washed with PBS buffer, and then stained with DAPI staining solution (Solarbio, China) for 5 min to observe fluorescence. The second-generation BMSCs were digested, centrifuged, and washed; 5-μL Annexin V-FITC and 5-μL PI (BD, USA) were added; and the cells were incubated at room temperature for 15 min in the dark. Cell apoptosis was detected by flow cytometry.

### Lentiviral transfection

According to the optimal transfection conditions (Eni.s + Polybrane) and optimal multiplicity of infection (MOI = 80) found in our pre-transfection experiments, the experimental group BMSCs were infected with a commercially available lentivirus (Shanghai Gene Chemical Co., Ltd., China) for 12 h, and a blank control group was established in parallel. After 4 days of culture, puromycin at 2 µg/ml was added to the blank control group and the experimental group. When all the cells in the blank control group died, the concentration of puromycin was reduced to 1 µg/ml, and a stable strain was finally selected.

### CHIP-qPCR

Chromatin was cross-linked with 37% formaldehyde, 1.25 M glycine was used to terminate cross-linking, and chromatin was cut by sonication. H3K27me3 (9733, Cell Signaling), HIF-1α (ab228649, Abcam), TF II (ab28179, Abcam), and RNA pol II (ab264350, Abcam)-specific antibodies were used for immunoprecipitation. DNA was extracted from the immunoprecipitates, and an SYBR-green mixture was used for the qPCR reaction. The relative expression of RNA was calculated using the ΔΔCt method. See Supplementary Table 1 for the primer sequences.

### Real-time quantitative PCR

Total RNA Extractor (Sangon Biotech, China) was used for RNA extraction, the M-MuLV First Strand cDNA Synthesis Kit (Sangon Biotech, China) was used for cDNA synthesis, and the SYBR-green mixture was used for qPCR reactions. The primer sequences were as follows: LncRNA Tmem235-F: CAGTGTTGTCAACTTCATCACTT; LncRNA Tmem235-R: TAGCTGGCTTTTTACCTCCTGG; EZH2-F: TGCCTCCTTAGACATTCTCCA; EZH2-R: AACCCATGTGTTTGGGTGGT; HIF-1α-F: CCTCTGGACTTGCCCCTTTC; HIF-1α-R: TCGACGTTCGGAACTCATCC; GAPDH-F: GACATGCCGCCTGGAGAAAC; GAPDH-R: AGCCCAGGATGCCCTTTAGT.

### Methylation-specific PCR

The extracted DNA was denatured with NaOH and mixed with hydroquinone and sodium bisulfite, and the modified DNA was eluted through a Wizerd DNA purification column, modified with NaOH, and precipitated with ethanol. The precipitated DNA was finally amplified by PCR. The primer sequences of the Tmem235 promoter amplified by PCR were as follows: methylated primer-F: TTGTGTTAAATGATGTAGAATTGAGC; methylated primer-R: TACCTATAAAAAAATCGAAATCGAA; unmethylated primer-F: TTTGTGTTAAATGATGTAGAATTGAGTG; unmethylated primer-R: TACCTATAAAAAAATCAAAAATCAAA.

### Immunofluorescence staining

After fixation and permeabilization, the BMSCs were divided into two groups. Group 1 received rabbit anti-EZH2 (ab191080, Abcam) at a 1/250 dilution, followed by goat anti-rabbit IgG (AlexaFluor® 488) (ab150077, Abcam) secondary antibody (green) at a 1/200 dilution. Group 2 received mouse anti-tri-methyl-histone H3(lys27) (ab6002, Abcam) at a 1/1000 dilution, followed by goat anti-mouse IgG H&L (Alexa Fluor® 594) (ab150120) at a 1/1000 dilution Resist (red). The nuclear counterstain was DAPI (blue).

### CRISPR/Cas9

Two knockout targets were designed at each position upstream and downstream of the EZH2 and HIF-1α genes to improve the knockout efficiency. Nested primers were designed downstream to test the knockout effect. Lentiviruses were produced by Shanghai Jikai Gene Chemical Technology Co. Ltd. The optimal transfection multiplicity and optimal transfection conditions were found in the pre-experiment, and Lv-Cas9-Sg EZH2, Lv-Cas9-Sg HIF-1α, and lentivirus negative control were transfected into BMSCs. Four days after transfection, an appropriate amount of puromycin was used to screen the mixed clone cell line. The non-mismatched enzyme method was used to test the knockout effect, and the designed nested primers were used to extract and amplify the genome of the mixed clone cell line, and a positive band indicated that the target was effective. After identifying positive bands, the monoclones were continually selected for subsequent experiments.

### Statistical analysis

All statistics were calculated and plotted using SPSS 19.0 and GraphPad Prism 9. The Shapiro–Wilk test was used to analyze data for normality. Student’s *t*-test was used for statistical significance analysis involving only two groups, and one-way ANOVA with Tukey’s post hoc test was used for statistical significance analysis involving more than two groups. *P* values < 0.05 were considered statistically significant.

## Results

### Hypoxia-induced decrease in the expression of LncRNA Tmem235 promotes BMSC apoptosis

**Fig. 1:**
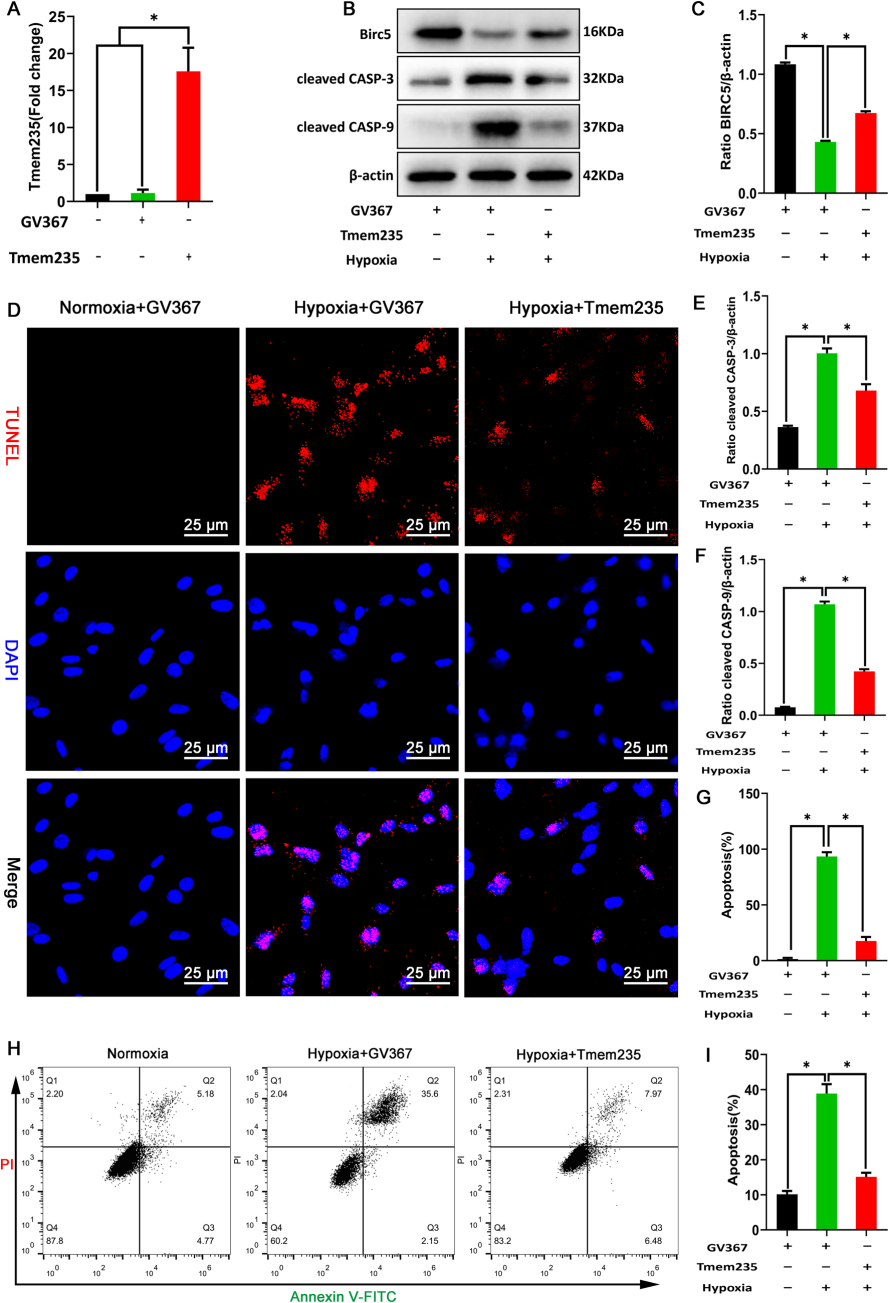
Long non-coding RNA transmembrane protein 235 (LncRNA Tmem235) inhibits hypoxic apoptosis of bone marrow mesenchymal stem cells (BMSCs). **A** Quantitative polymerase chain reaction (qPCR) detection of LncRNA Tmem235 overexpression (n = 5); empty vector (GV367). **B**, **C** and **E**, **F** Western blot to detect the expression levels of baculoviral inhibitor of apoptosis [IAP] repeat-containing 5 (BIRC5), cleaved caspase-3 (CASP-3), and cleaved caspase-9 (CASP-9) (n = 3). **D** and **G** Apoptosis detected by TUNEL/DAPI (n = 5); terminal deoxynucleotidyl transferase deoxyuridine-5′-triphosphate nick-end labeling (TUNEL), 4′,6-diamidino-2-phenylindole (DAPI). **H**–**I** Apoptosis detected by Annexin V-FITC/PI (n = 5); fluorescein isothiocyanate (FITC), propidium iodide (PI). In **A**, **C**, **E**–**G**, and **I**, data are presented as the mean ± standard deviation (SD); statistical significance was calculated using one-way ANOVA with Tukey’s post hoc test.^*^P < 0.05

Through adipogenic, osteogenic, and chondrogenic induction and surface antigen identification of BMSCs isolated and cultured by whole bone marrow adherence, the isolated and cultured BMSCs were found to have typical morphological characteristics, growth patterns, surface markers, and multi-directional differentiation potential (Supplementary Fig. 1A–E). After BMSCs were treated with hypoxia at different oxygen concentrations for 48 h, the mitochondrial membrane potential and ATP content gradually decreased with the increase in the degree of hypoxia (Supplementary Fig. 2A–C), while the ROS content gradually increased (Supplementary Fig. 2E–F). The expression of BIRC5 was gradually down-regulated, while the expressions of cleaved CASP-3 and cleaved CASP-9 were gradually up-regulated (Supplementary Fig. 2G–J). The apoptosis rate of BMSCs increased gradually (Supplementary Fig. 2 K–L), while the results of qPCR showed a gradual decrease in LncRNA Tmem235 expression (Supplementary Fig. 2D). Our previous studies have demonstrated the critical role of LncRNA Tmem235 during hypoxia-induced apoptosis in BMSCs. Briefly, we transfected BMSCs with LncRNA Tmem235-overexpressing lentivirus to overexpress LncRNA Tmem235 (Fig. 1A), followed by hypoxia-treated BMSCs. The results showed that overexpression of LncRNA Tmem235 increased BIRC5 expression, while the expression of cleaved CASP-9 and cleaved CASP-3 decreased (Fig. 1B, C, E, F), as did the rate of BMSC apoptosis (Fig. 1D, G–I). In conclusion, the hypoxia-induced decrease in the expression of LncRNA Tmem235 promotes BMSC apoptosis.

### Hypoxia induces the LncRNA Tmem235 promoter H3K27me3 to inhibit LncRNA Tmem235 expression

**Fig. 2:**
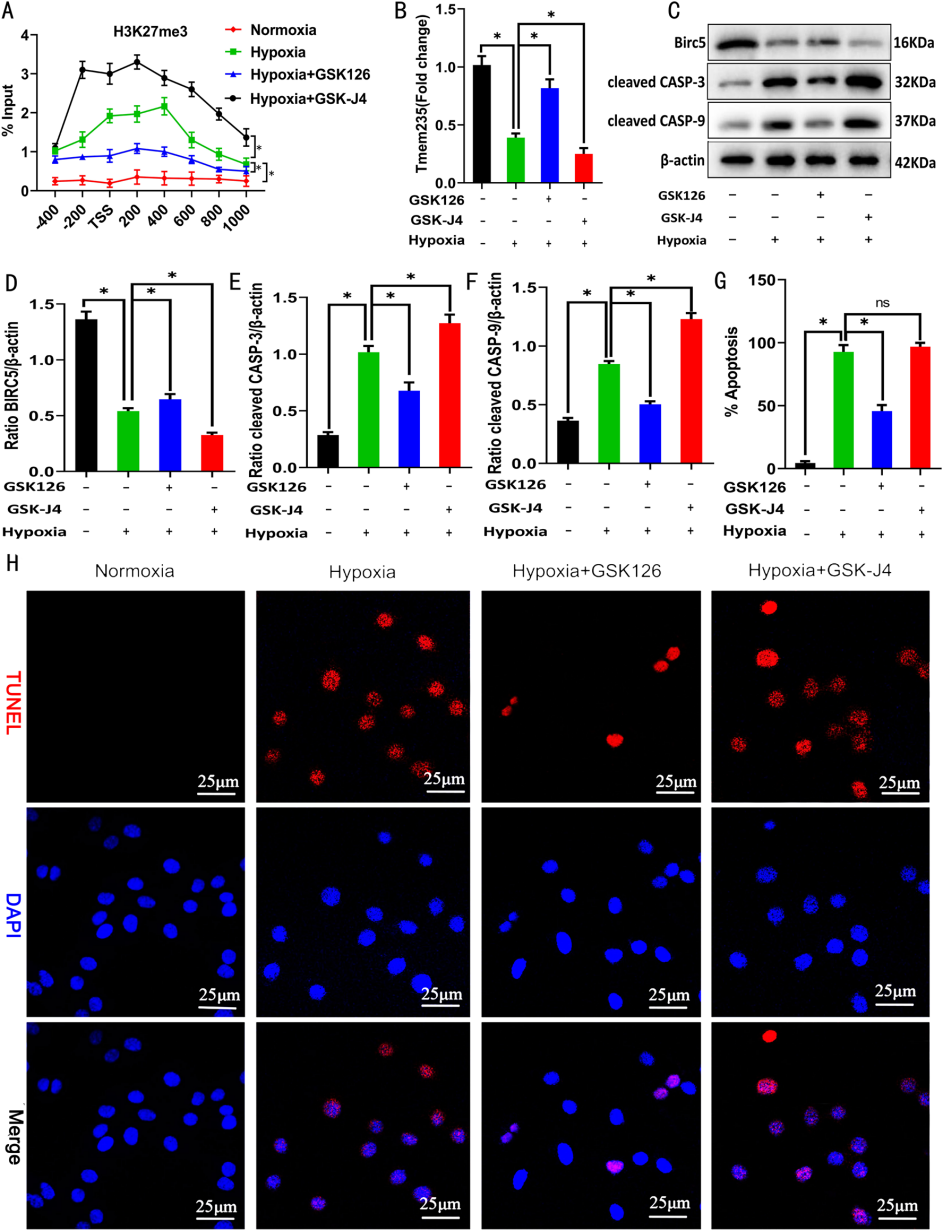
Relationship between the modification of trimethylation of histone H3 lysine 27 (H3K27me3) in the promoter region of LncRNA Tmem235 and the hypoxic apoptosis of BMSCs. **A** Chromatin immunoprecipitation-qPCR (CHIP-qPCR) to detect the level of H3K27me3 in the promoter region of LncRNA Tmem235 (n = 3); transcription start site (TSS), EZH2 inhibitor (GSK126), H3K27 demethylase inhibitor (GSK-J4). **B** qPCR to detect the expression of LncRNA Tmem235 (n = 5). **C**–**F** Western blot to detect the expression levels of BIRC5, cleaved CASP-3, and cleaved CASP-9 (n = 3). **G**–**H** TUNEL/DAPI to detect cell apoptosis (n = 6). In **A**, **B** and **D**–**G**, data are presented as the mean ± standard deviation (SD); statistical significance was calculated using one-way ANOVA with Tukey’s post hoc test. ^*^P < 0.05, ^ns^*P* > 0.05

Following the treatment of BMSCs with hypoxia for 48 h, CHIP-qPCR showed that the level of LncRNA Tmem235 promoter H3K27me3 modification was increased (Fig. 2A). We next treated BMSCs with the H3K27 methyltransferase EZH2 inhibitor. Next, we subjected BMSCs to hypoxia and found that the expression of LncRNA Tmem235 and BIRC5 increased (Fig. 2B), the expression of cleaved CASP-9 and cleaved CASP-3 decreased (Fig. 2C–F), and the hypoxic apoptosis of BMSCs decreased (Fig. 2G–H). Conversely, BMSCs were treated with an inhibitor of H3K27 demethylase KDM6 (GSK-J4) to elevate the level of H3K27me3 in the promoter region of LncRNA Tmem235 (Fig. 2A). The hypoxia treatment of BMSCs decreased the expression of LncRNA Tmem235 and BIRC5 (Fig. 2B), increased the activities of CASP-9 and CASP-3 (Fig. 2C–F), and increased the hypoxic apoptosis of BMSCs (Fig. 2G–H). The above results indicate that hypoxia-induced downregulation of LncRNA Tmem235 expression was related to an increased level of H3K27me3 modification in the LncRNA Tmem235 promoter.

### LncRNA Tmem235 promoter H3K27me3 cooperates with DNA methylation to inhibit the expression of LncRNA Tmem235

**Fig. 3:**
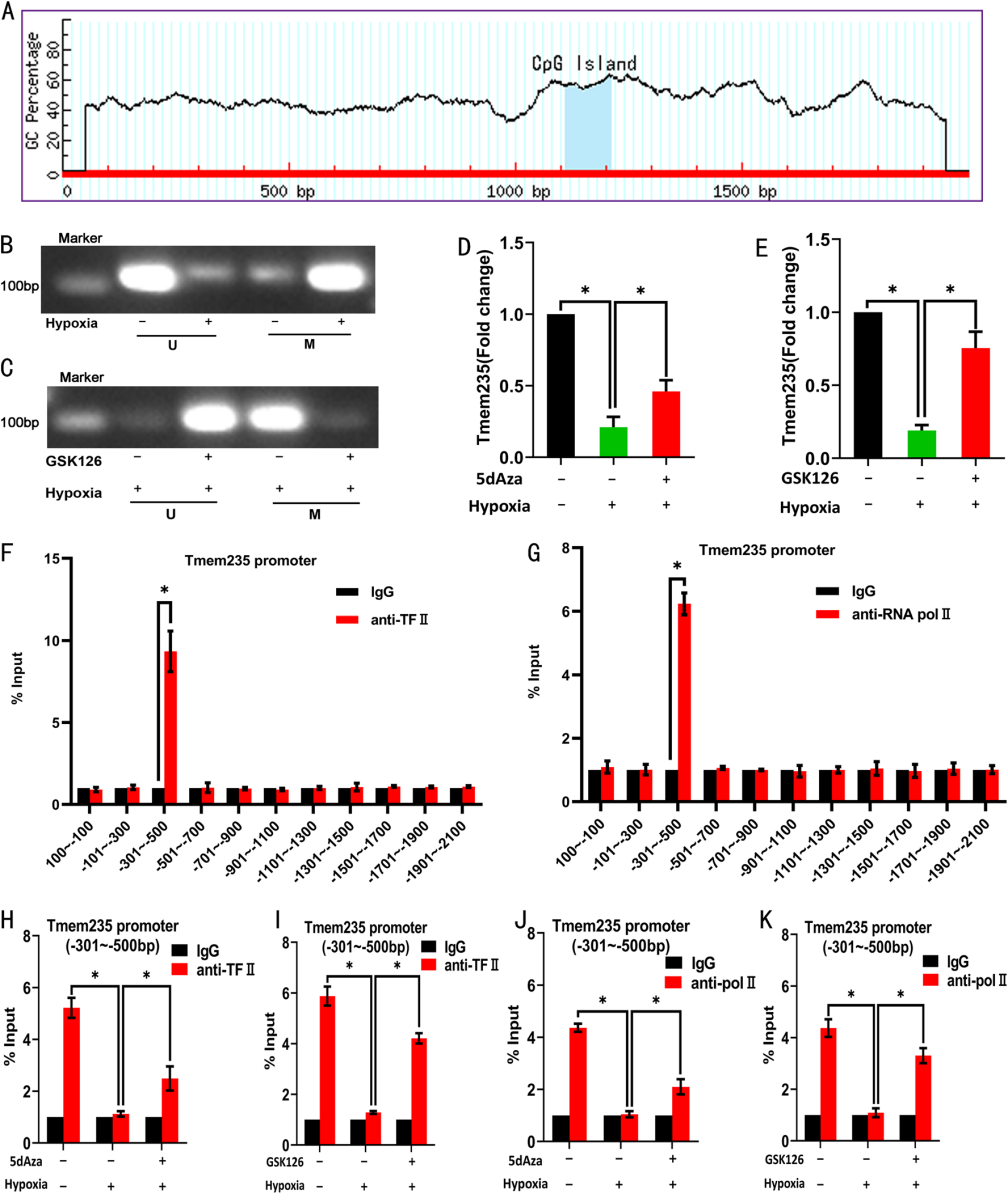
H3K27me3 modification in the promoter region of LncRNA Tmem235 cooperates with DNA methylation to inhibit the expression of LncRNA Tmem235. **A** MethPrimer database predicts CpG islands in the promoter region of LncRNA Tmem235. **B**–**C** Detection of methylation levels in the promoter region of LncRNA Tmem235 by methylation-specific PCR (n = 4); unmethylation primer (U), methylation primer (M). **D**–**E** qPCR to detect the expression of LncRNA Tmem235 (n = 5); 5-aza-2ʹ-deoxycytidine (5dAza). **F**–**K** CHIP-qPCR detection of the enrichment levels of transcription factor II (TF II) and RNA polymerase II (pol II) in the promoter region of LncRNA Tmem235 (n = 5). In **D**–**K**, data are presented as the mean ± standard deviation (SD); statistical significance was calculated using Student’s *t*-test in **F** and **G** and one-way ANOVA with Tukey’s post hoc test in **D**, **E**, and **H**–**K** ^*^P < 0.05

DNA methylation plays an important role in controlling gene expression and mainly occurs in CpG islands of gene promoters. Using bioinformatics analysis, we predicted that the LncRNA Tmem235 promoter contains an independent CpG island (GC Percent > 50.0, Obs/Exp > 0.60) (Fig. 3A), indicating that the LncRNA Tmem235 promoter can be methylated to affect gene expression. Methylation-specific PCR revealed that LncRNA Tmem235 promoter DNA methylation was up-regulated in hypoxia-treated BMSCs (Fig. 3B). Treatment with the H3K27 methyltransferase EZH2 inhibitor (GSK126) reduced the H3K27me3 modification level of the LncRNA Tmem235 promoter, and the DNA methylation level of LncRNA Tmem235 promoter was down-regulated accordingly (Fig. 3C). Finally, we treated BMSCs with DNA methylation inhibitors 5-aza-2'-deoxycytidine (5-dAza) and GSK126 for 4 days and then treated BMSCs with hypoxia. The results showed that the expression of LncRNA Tmem235 was up-regulated (Fig. 3D). CHIP-qPCR showed that transcription factor II (TF II) and RNA polymerase II (pol II) were significantly enriched in the promoter region of LncRNA Tmem235 (Fig. 3F–K). In conclusion, in hypoxia-treated BMSCs, the LncRNA Tmem235 promoter H3K27me3 cooperates with DNA methylation to inhibit LncRNA Tmem235 expression.

### EZH2 mediates the modification of the LncRNA Tmem235 promoter H3K27me3

**Fig. 4:**
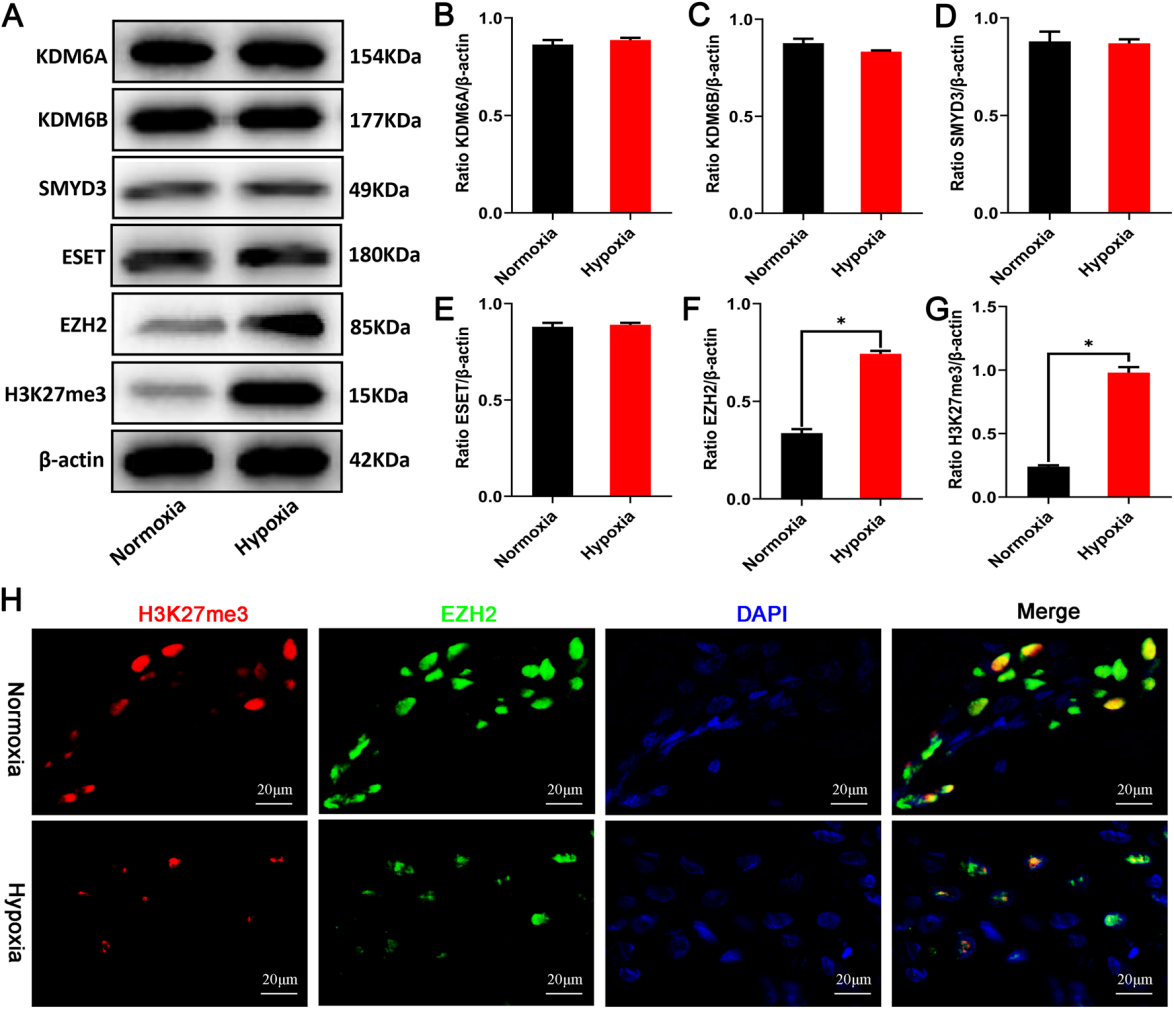

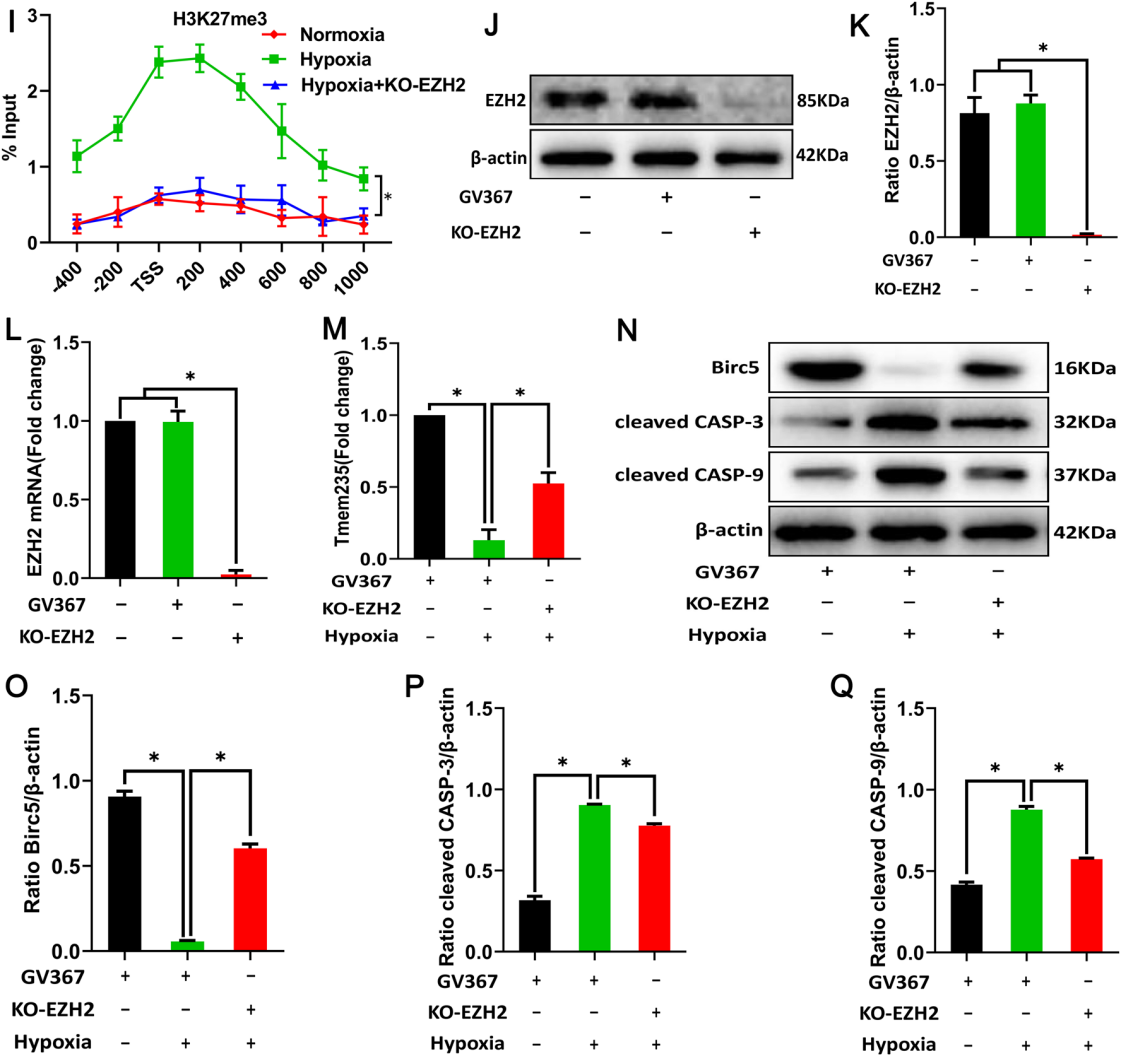
Enhancer of zeste homolog 2 (EZH2) promotes the modification of H3K27me3 in the promoter region of LncRNA Tmem235. **A**–**G** Western blot detection of the expression levels of KDM6A, KDM6B, SMYD3, ESET, EZH2, and H3K27me3 (n = 3); lysine demethylase 6 (KDM6), SET and MYND domain-containing protein 3 (SMYD3), ERG-associated protein with the SET domain (ESET). **H** Immunofluorescence detection of the common nuclear localization of H3K27me3 and EZH2 (n = 4). **I** CHIP-qPCR detection of the H3K27me3 level in the promoter region of LncRNA Tmem235 (n = 3); knockout (KO). **J**–**K** Western blot detection of the EZH2 expression level (n = 3). **L** qPCR detection of EZH2 expression (n = 5). **M** qPCR to detect the expression of LncRNA Tmem235 (n = 5). **N**–**Q** Western blot to detect the expression levels of BIRC5, cleaved CASP-3, and cleaved CASP-9 (n = 3). In **B**–**G**, **I**, **K**–**M**, and **O**–**Q**, data are presented as the mean ± standard deviation (SD); statistical significance was calculated using Student’s *t*-test in **B**–**G** and one-way ANOVA with Tukey’s post hoc test in **I**, **K**–**M**, and **O**–**Q** ^*^P < 0.05

Under the action of histone methylases (EZH2, SMYD3, and ESET) and histone demethylases (KDM6A, KDM6B/JMJD3), the methylation modification and demethylation modification of H3K27 maintain a dynamic balance. The expression of the above enzymes will be affected by the action of external factors, such as hypoxia or drugs, thereby affecting the methylation modification of H3K27 [31–33]. The expression of the demethylases KDM6A and KDM6B/JMJD3 and the methylases SMYD3 and ESET did not change significantly following hypoxia treatment of BMSCs, while the expression of methylase EZH2 was significantly up-regulated and the expression of H3K27me3 was increased (Fig. 4A–G). Additionally, immunofluorescence also showed that hypoxia treatment of BMSCs increased the co-localization of EZH2 and H3K27me3 in the cell nucleus (Fig. 4H). In hypoxia-treated BMSCs, knockdown of the EZH2 gene (Fig. 4J–L) decreased the LncRNA Tmem235 promoter H3K27me3 modification level (Fig. 4I), up-regulated the expression of LncRNA Tmem235 (Fig. 4M) and BIRC5, and decreased the expression of cleaved CASP-9 and cleaved CASP-3 (Fig. 4N–Q). The above data indicated that under hypoxic conditions, overexpressed EZH2 promotes the modification of the LncRNA Tmem235 promoter H3K27me3 and finally inhibits the expression of LncRNA Tmem235, thereby affecting the hypoxic apoptosis of BMSCs.

### Hypoxia regulates modification of the LncRNA Tmem235 promoter H3K27me3 through the HIF-1a/EZH2 signaling pathway to promote hypoxic apoptosis of BMSCs

**Fig. 5:**
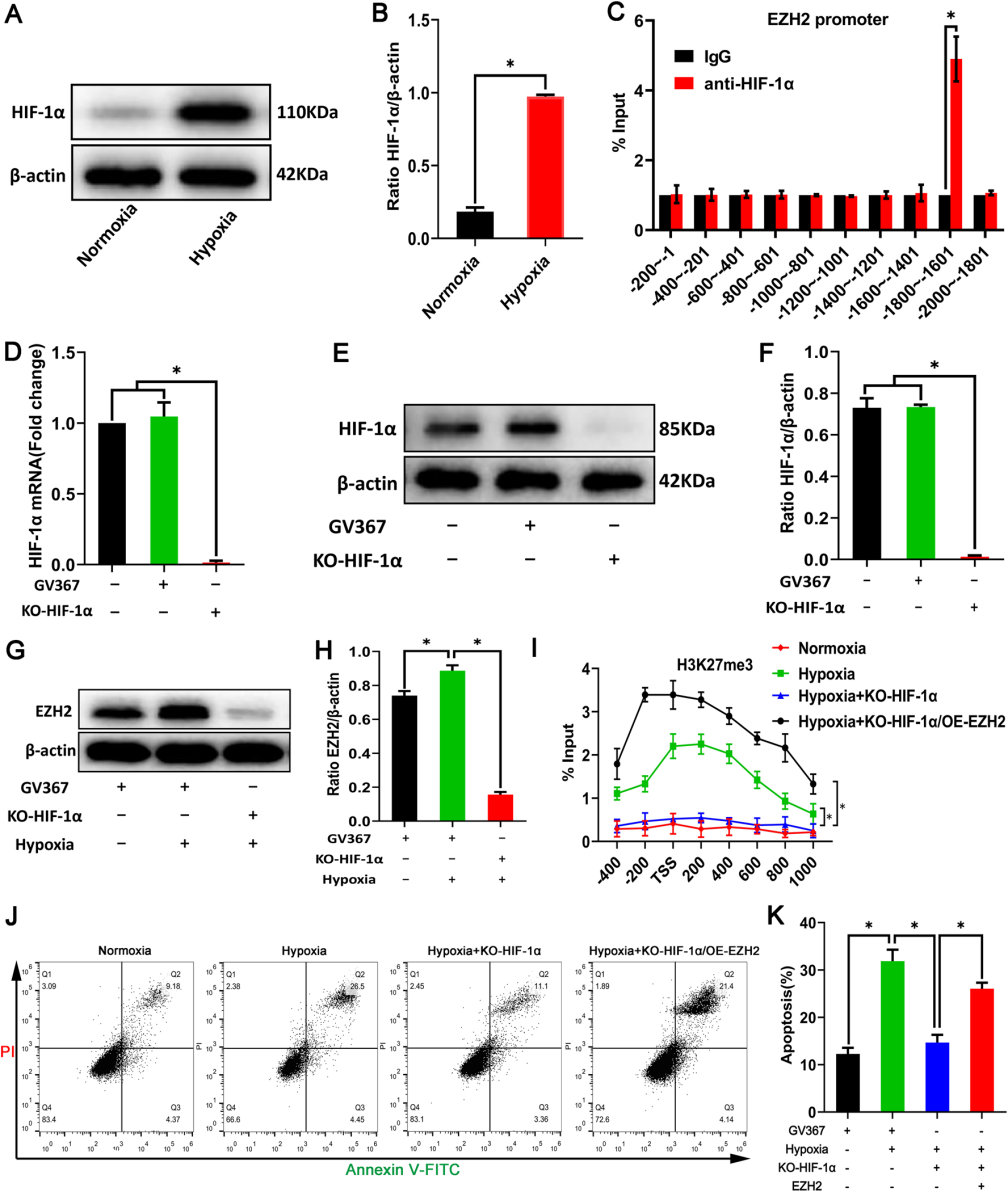
Hypoxia regulates LncRNA Tmem235 promoter H3K27me3 modification through the hypoxia-inducible factor-1alpha (HIF-1a)/EZH2 signaling pathway to promote hypoxic apoptosis of BMSCs. **A**, **B** Western blot detection of the HIF-1α expression level (n = 3). **C** Detection of the enrichment level of HIF-1α in the promoter region of EZH2 by CHIP-qPCR (n = 5). **G**–**H** Western blot detection of the expression level of EZH2 (n = 3). **I** CHIP-qPCR detects the level of H3K27me3 in the promoter region of LncRNA Tmem235 (n = 3). **J**–**K** Apoptosis was detected by Annexin V-FITC/PI (n = 5); overexpression (OE). In **B**–**D**, **F**, **H**, **I**, and **K**, data are presented as the mean ± standard deviation (SD); statistical significance was calculated using Student’s *t*-test in **B**–**C** and one-way ANOVA with Tukey’s post hoc test in **D**, **F**, **H**–**I**, and **K** ^*^P < 0.05

As a hypoxic transcriptional activator, HIF-1α can bind to the gene promoter HRE to activate gene expression, while the EZH2 promoter region contains HRE [28–30]. Therefore, we speculate that hypoxia may activate EZH2 gene expression through HIF-1α, and then, EZH2 mediates the modification of LncRNA Tmem235 promoter H3K27me3 to promote hypoxic apoptosis of BMSCs. To test this conjecture, we first found that HIF-1α expression was up-regulated in hypoxia-treated BMSCs (Fig. 5A–B). Then, we predicted the existence of a binding site (GTAAGTGC) between HIF-1α and the EZH2 promoter region through the NCBI and JASPAR databases. CHIP-qPCR further showed that there was an interaction between HIF-1α and the EZH2 promoter (–1800 to –1601 bp) (Fig. 5C). Finally, we used CRISPR/Cas9 technology to knock out HIF-1α (KO-HIF-1α) (Fig. 5D–F). Following hypoxia treatment of BMSCs, we found that the expression of EZH2 decreased (Fig. 5G–H), LncRNA Tmem235 promoter H3K27me3 modification level decreased (Fig. 5I), and the apoptosis of BMSCs decreased (Fig. 5J–K). In addition, on the basis of knocking down HIF-1α and overexpressing EZH2, it was found that the LncRNA Tmem235 promoter H3K27me3 modification level (Fig. 5I) and BMSC apoptosis rate (Fig. 5J–K) were both increased.

These results suggest that hypoxia promotes the modification of the LncRNA Tmem235 promoter H3K27me3 through the HIF-1α/EZH2 signaling pathway, ultimately leading to hypoxic apoptosis of BMSCs.

## Discussion

BMSCs have shown good application prospects for treating clinical diseases [34–36]. However, the transplanted BMSCs have high rates of apoptosis in the hypoxic environment of the lesion area, which greatly limits the transplantation efficacy of BMSCs [7]. Therefore, identifying new targets and exploring the mechanism of hypoxic apoptosis of BMSCs are of great practical significance for inhibiting hypoxic apoptosis of BMSCs. In this study, we found that hypoxia increased the level of H3K27me3 modification of the LncRNA Tmem235 promoter by activating the HIF-1α/EZH2 signaling pathway and inhibiting the expression of LncRNA Tmem235, thereby leading to hypoxic apoptosis of BMSCs.

Although LncRNA cannot directly encode proteins, it can regulate gene expression in various ways [37, 38], which can affect apoptosis. Indeed, Fan et al. [39] found that LncRNA MALAT1 could activate AMP-activated protein kinase (AMPK) signaling by inhibiting the expression of protein phosphatase Mg^2+^/Mn^2+^-dependent 1e (Ppm1e), reducing ROS production and inhibiting apoptosis. Additionally, Han et al. [40] found that silencing LncRNA LINC00857 could promote cancer cell apoptosis by inhibiting the expression of BIRC5. Our study revealed that the expression of LncRNA Tmem235 was decreased in hypoxia-treated BMSCs, while the hypoxic apoptosis of BMSCs was reduced after overexpression of LncRNA Tmem235. These findings suggest that LncRNA Tmem235 can inhibit the hypoxic apoptosis of BMSCs, which provides a more theoretical basis for targeting LncRNA to inhibit the hypoxic apoptosis of BMSCs.

In hypoxia-treated BMSCs, we identified that the LncRNA Tmem235 promoter H3K27me3 cooperated with LncRNA Tmem235 promoter methylation to suppress LncRNA Tmem235 expression. Under hypoxic conditions, both the H3K27me3 modification of the LncRNA Tmem235 promoter and the methylation level of the LncRNA Tmem235 promoter were significantly increased, and when the LncRNA Tmem235 promoter H3K27me3 modification level decreased, the DNA methylation of the LncRNA Tmem235 promoter also decreased. Delphine Douillet et al. [25] found that in the case of mixed lineage leukemia (MLL2) knockout, both H3K27me3 and DNA methylation inhibited the expression of Magohb (the most significantly downregulated gene in the absence of MLL2) but that H3K27me3 predominated in repressing Magohb gene expression relative to DNA methylation. Additionally, they demonstrated that H3K27me3 repression of gene expression could be rescued by gene demethylation. Brinkman et al. [24, 41] also found that the deposition of H3K27me3 could be affected by the treatment of cells with 5dAza; that is, the decrease in DNA methylation could inhibit the modification of H3K27me3. In summary, H3K27me3 modification and DNA methylation can interact and influence each other to jointly regulate gene expression.

EZH2, as a master regulator, causes chromatin heterochromatinization by promoting H3K27 methylation modification, which serves to deactivate many genes [42]. Jadhav U et al. [43] found that EZH1 and EZH2 can compensate for each other in H3K27me3. In the absence of EZH2, EZH1 partially compensates for the effect of EZH2, thereby enhancing H3K27me3 modification. After knocking out EZH2 and treating BMSCs with hypoxia, we found that the modification level of the LncRNA Tmem235 promoter H3K27me3 was decreased, and the expression of LncRNA Tmem235 was increased, indicating that in hypoxia-treated BMSCs, the partial compensatory effect of EZH1 may partially increase the level of H3K27me3 modification of the LncRNA Tmem235 promoter after EZH2 knockout, but it is not sufficient to maintain gene silencing.

We also found that the ROS content and the level of HIF-1α expression were increased in hypoxia-treated BMSCs. HIF-1α can bind to the promoter of EZH2, thereby activating the expression of EZH2. In mammalian cells, HIF-1α is also ubiquitously expressed in non-hypoxic states, but synthetic HIF-1α is rapidly degraded by the intracellular oxygen-dependent ubiquitin protease degradation pathway. HIF-1α can be stably expressed under hypoxic conditions. Under hypoxic conditions, the increase in intracellular ROS will activate the poly[ADP-ribose] polymerase 1 (PARP-1) to induce poly-ADP ribosylation modification (PARylation) of the C-ter-specific residues in the two regions, thereby improving the stability and activity of HIF-1α [44], such that HIF-1α is not completely degraded. In the absence of PARP-1, the binding of HIF-1α to DNA is located in a shorter HRE-like sequence [24], which results in reduced binding, that is, under hypoxic conditions, activated PARP-1 not only regulates the expression of HIF-1α but also the binding of HIF-1α and EZH2 genes to affect the expression of EZH2. Chen [45] found that under hypoxia, HIF-1α could upregulate TWIST to promote EZH2 overexpression, thereby regulating the expression of related genes. In addition, Chang [46] found that in breast cancer, HIF-1α promotes EZH2 transcription and upregulates EZH2 expression by binding to HRE in the EZH2 promoter region. The above results indicate that HIF-1α is a key upstream molecule regulating EZH2 expression.

**Fig. 6:**
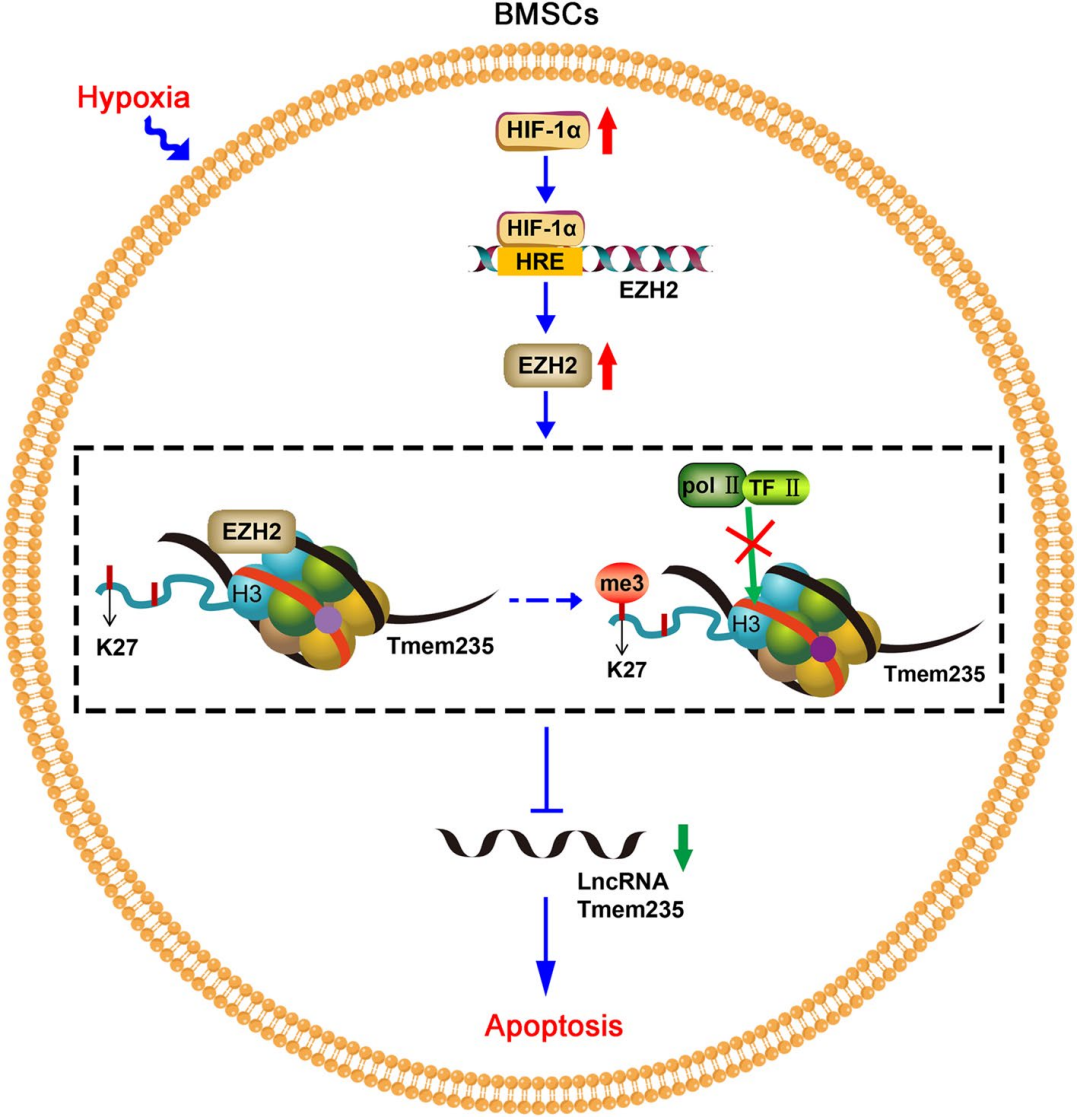
Mechanistic model outlining how the hypoxia-induced LncRNA Tmem235 promoter H3K27me3 promotes apoptosis in BMSCs. HIF-1α expression was increased in BMSCs under hypoxia. As a hypoxic transcriptional activator, HIF-1α binds to the hypoxia responsive element (HRE) of the EZH2 promoter and promotes the expression of EZH2. Under the action of EZH2, H3K27 in the promoter region of LncRNA Tmem235 (red band) undergoes trimethylation (me3) modification, while the CpG island (light purple circle) in the promoter region of LncRNA Tmem235 undergoes methylation simultaneously (dark purple circle). H3K27me3 cooperates with DNA methylation to inhibit the binding of TF II and pol II to the LncRNA Tmem235 promoter, thereby inhibiting the expression of LncRNA Tmem235 and promoting the hypoxic apoptosis of BMSCs

In conclusion, in hypoxia-treated BMSCs, we found that overexpressed HIF-1α inhibited LncRNA Tmem235 expression by activating EZH2 expression, which in turn increased LncRNA Tmem235 promoter H3K27me3 modification and synergized with LncRNA Tmem235 promoter DNA methylation to inhibit LncRNA Tmem235 expression and promote BMSC Hypoxic apoptosis (Fig. 6).

## Supplementary Information

Below is the link to the electronic supplementary material.

Supplementary file1 (DOCX 274 kb)

Supplementary file2 (JPG 6640 kb)

Supplementary file3 (JPG 9769 kb)

## Data Availability

All data included in this study are available from the corresponding author.

## Abbreviations

BMSCs: Bone marrow mesenchymal stem cells
ROS: Reactive oxygen species
LncRNA: Long non-coding RNA
LncRNA Tmem235: LncRNA transmembrane protein 235
BIRC5: Baculoviral inhibitor of apoptosis [IAP] repeat-containing 5
miR-34a-3p: MicroRNA 34a-3p
CASP-3/9: Caspase-3/9
H3K27me3: Trimethylation of histone H3 lysine 27
PRC2: Polycomb repressive complex 2
EZH2: Enhancer of zeste homolog 2
KDM6: Lysine demethylase 6
SMYD3: SET and MYND domain-containing protein 3
ESET: ERG-associated protein with the SET domain
HIF-1α: Hypoxia-inducible factor-1alpha
HRE: Hypoxia-responsive element
CHIP-qPCR: Chromatin immunoprecipitation-quantitative real-time PCR
TF II: Transcription factor II
pol II: RNA polymerase II
Ppm1e: Protein phosphatase Mg^2+^/Mn^2+^dependent 1e
AMPK: AMP-activated protein kinase
MLL2: Mixed lineage leukemia
PARP-1: Poly (ADP-ribose) polymerase
C-ter: C-terminus
PARylation: Poly-ADP ribosylation modification
5dAza: 5-Aza-2ʹ-deoxycytidine
