# Supplementary figures and images for: Hypoxic condition induced H3K27me3 modification of the LncRNA Tmem235 promoter thus supporting apoptosis of BMSCs
Source: Apoptosis. 2022 Jul 2;27(9-10):762–77. doi: 10.1007/s10495-022-01747-8 (PMC9482900; doi:10.1007/s10495-022-01747-8)

**Supplementary Table 1. Primer sequences**

**
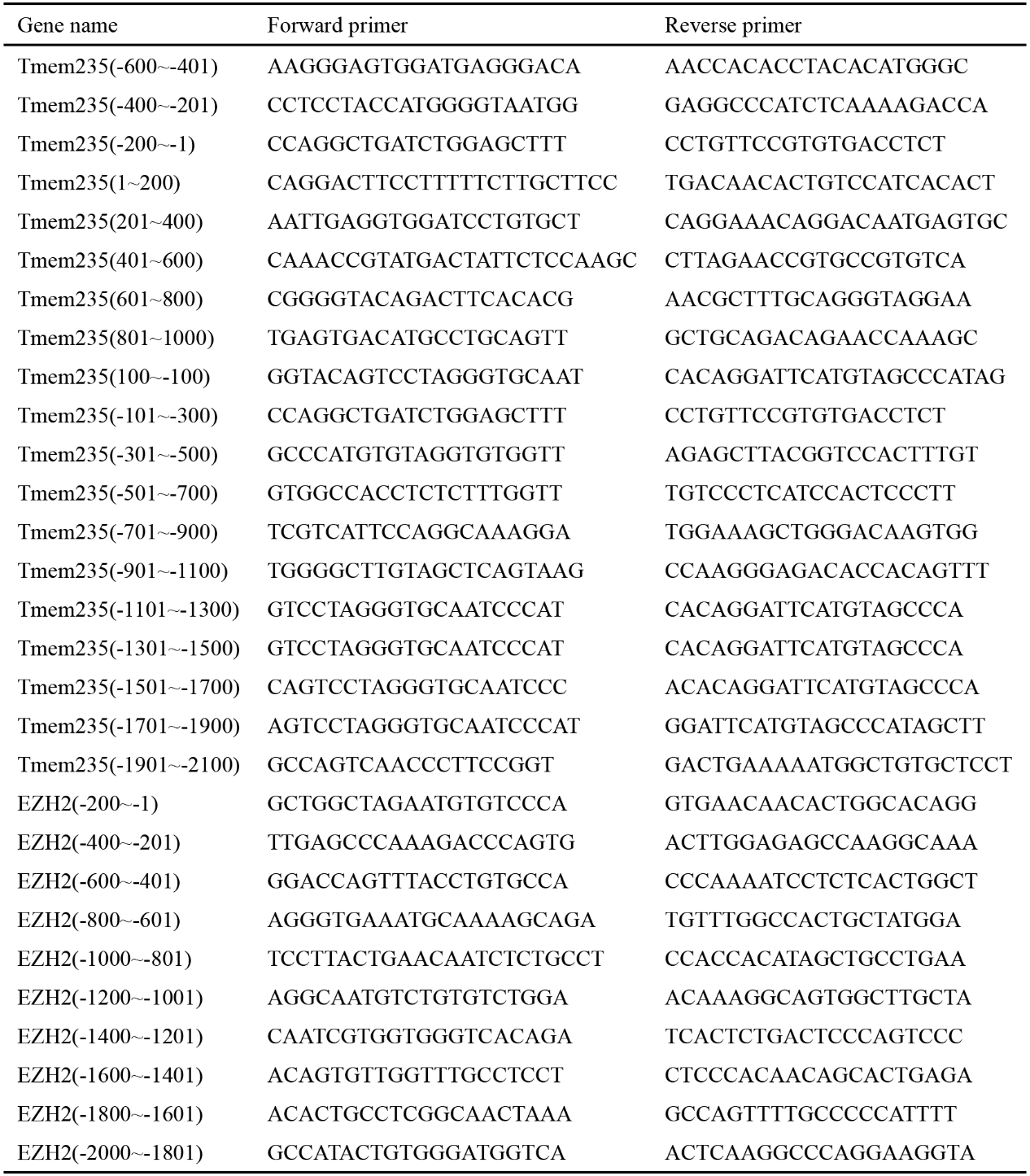
**

Supplement: Supplementary file 1 — Supplementary file1 (DOCX 274 kb) [file 10495_2022_1747_MOESM1_ESM.docx]

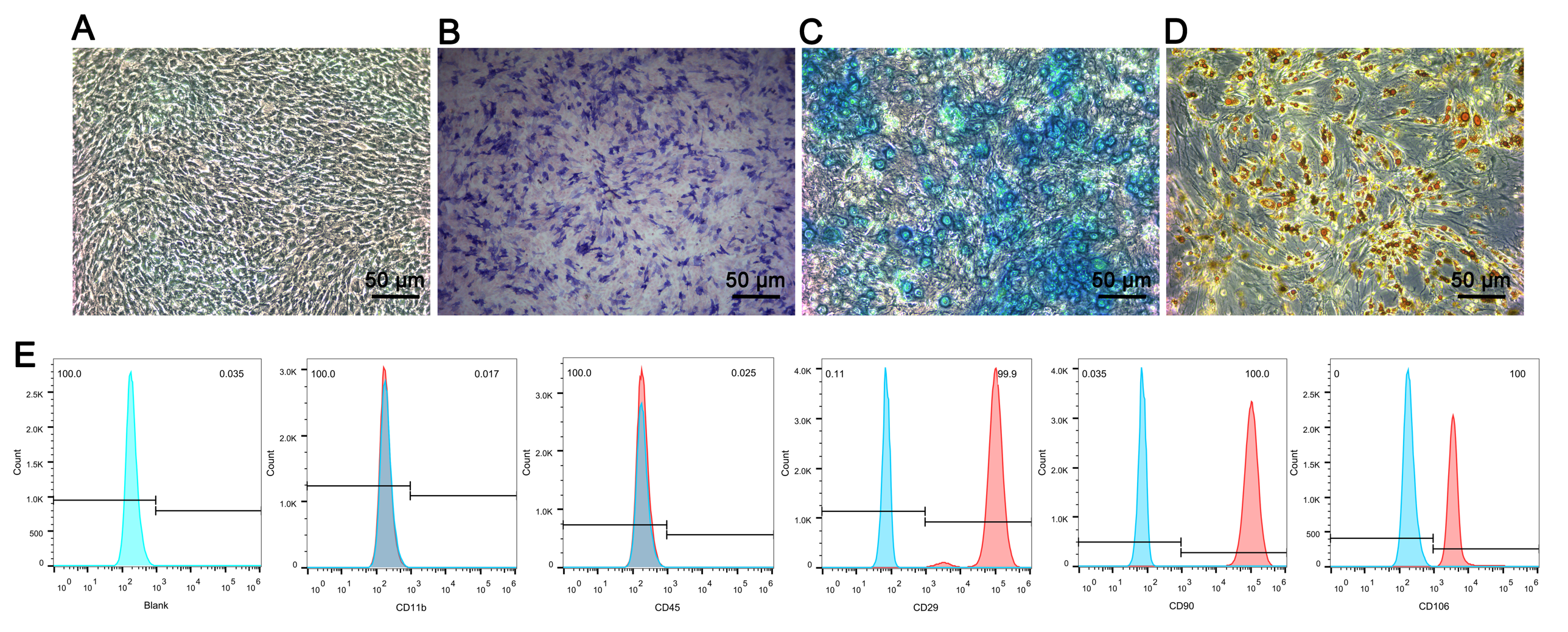

Supplement: Supplementary file 2 — Supplementary file2 (JPG 6640 kb) [file 10495_2022_1747_MOESM2_ESM.jpg]
